# Air pollution and refraining from visiting health facilities: a cross-sectional study of domestic migrants in China

**DOI:** 10.1186/s12889-022-14401-4

**Published:** 2022-11-02

**Authors:** Zhixin Liu, Chaojie Liu, Yu Cui, Junping Liu, Huanyu Zhang, Yajie Feng, Nan Wang, Mingli Jiao, Zheng Kang, Xiaoxue Xu, Juan Zhao, Chen Wang, Dandan Zou, Libo Liang, Qunhong Wu, Yanhua Hao

**Affiliations:** 1 Department of Social Medicine, School of Health Management, Harbin Medical University, 150081 Harbin, China; 2 Department of Public Health, School of Psychology and Public Health, La Trobe University, 3086 Melbourne, VIC Australia; 3 Department of Health Economics, School of Health Management, Harbin Medical University, 150081 Harbin, China; 4 Southwest Hospital, Third Military Medical University (Army Medical University), 400000 Chongqing, China; 5 Xinqiao Hospital, Third Military Medical University (Army Medical University), 400037 Chongqing, China

**Keywords:** Air pollution, Refrain from visiting health facilities, Domestic migrants, China

## Abstract

**Background:**

Local environmental factors are associated with health and healthcare-seeking behaviors. However, there is a paucity in the literature documenting the link between air pollution and healthcare-seeking behaviors. This study aimed to address the gap in the literature through a cross-sectional study of domestic migrants in China.

**Methods:**

Data were extracted from the 2017 China Migrants Dynamic Survey (*n* = 10,051) and linked to the official air pollution indicators measured by particulate matter (PM_2.5_ and PM_10_) and air quality index (AQI) in the residential municipalities (*n* = 310) of the study participants over the survey period. Probit regression models were established to determine the association between air pollution and refraining from visiting health facilities after adjustment for variations in the predisposing, enabling and needs factors. Thermal inversion intensity was adopted as an instrumental variable to overcome potential endogeneity.

**Results:**

One unit (µg/m^3^) increase in monthly average PM_2.5_ was associated with 1.8% increase in the probability of refraining from visiting health facilities. The direction and significance of the link remained unchanged when PM_2.5_ was replaced by AQI or PM_10_. Higher probability of refraining from visiting health facilities was also associated with overwork (β = 0.066, *p* = 0.041) and good self-related health (β = 0.171, *p* = 0.006); whereas, lower probability of refraining from visiting health facilities was associated with short-distance (inter-county) migration (β=-0.085, *p* = 0.048), exposure to health education (β=-0.142, *p* < 0.001), a high sense of local belonging (β=-0.082, *p* = 0.018), and having hypertension/diabetes (β=-0.169, *p* = 0.005).

**Conclusion:**

Air pollution is a significant predictor of refraining from visiting health facilities in domestic migrants in China.

**Supplementary Information:**

The online version contains supplementary material available at 10.1186/s12889-022-14401-4.

## Background

With the acceleration of globalization and rapid socioeconomic development in China, domestic migrants have become a powerful driving force of China’s industrialization and urbanization. However, increased population mobility, in particular rural-to-urban migration, has also imposed a great challenge to the provision of healthcare services [1, 2]. Like in many other countries, there have been increasing concerns in China about the inequality of healthcare services between the migrants and their local counterparts [3]. Overall, the migrants are less likely to use healthcare services than the non-migrants [4], in particular in those who are not able to bring their social health insurance entitlements to the migration destinations. This is despite the fact that migrants usually suffer more health problems than non-migrants due to poor living conditions [5], limited health literacy [6], overwork [7], and a lack of social support [8]. The exposure to a changing environment can add additional risks to the physical and mental health of the migrants [9].

Massive population migration is also associated with a substantial increase in PM_2.5_ emissions [2]. According to the 2017 China Environmental Bulletin (https://www.mee.gov.cn/hjzl/zghjzkgb/lnzghjzkgb/), 239 (70.7%) of the 338 municipalities in China failed to meet all of the governmental air quality standards measured by SO_2_, NO_2_, PM_10_, PM_2.5_, CO, and O_3_. On average, 22% days in a year recorded an air quality index (AQI) below the standards. In China, PM_2.5_ is the main air pollutant, which has demonstrated harmful effects on the cardiovascular and respiratory systems [10, 11]. Compared with windblown soil dusts, PM_2.5_ contains more toxic substances like acids and heavy metals, which can penetrate deeper into the lungs of human being [12]. Long-term exposure to PM_2.5_ can increase the incidence of hypertension, respiratory diseases, cardiovascular diseases, and neurological diseases [13–15].

Industrial pollution has been widely perceived as a sizeable health risk by the public [16]. Children and old people are particularly vulnerable to pollution [17]. Air pollution is not only associated with physical health harm, but also negative emotion and mental health problems, such as anxiety [18]. Exposure to air pollution affects the brain function, which can trigger behavioral changes [19, 20]. There has been increasing awareness of the harm of air pollution among the public. People respond to the problem in a variety of ways. Szyszkowicz reported that air pollution is associated with increased substance abuse [21]. Chew and colleagues classified individual coping strategies for air pollution into risk aversion, ambiguity aversion, and time decision impatience [22]. These coping strategies can trigger changes in health behaviors and healthcare seeking behaviors. For example, Yu et al. in a study of school students in China found that air pollution discourages physical activities [23]. Neidell reported that people living with asthma may reduce outdoor exposure when they perceive heavy air pollution [24]. Previous studies have been focused on the impact of air pollution on healthcare services relating to respiratory diseases. However, air pollution is also strongly associated with increased cardiovascular morbidity and mortality [17]. There has been a paucity in the literature documenting the extent of general patients to refrain from seeking healthcare due to concerns of air pollution. Empirical evidence shows that refraining from healthcare can cause serious harm to patients [25], decreasing quality of life [4, 26]. It disproportionally impacts people with low socioeconomic status, exacerbating inequity in health and healthcare. Both personal idiosyncrasies and environmental factors play a role in individual decisions of seeking (or refraining from seeking) healthcare services [27].

This study aimed to address the gap in the literature by determining the incidence of refraining from visiting health facilities in the domestic migrant population in China and its association with air pollution. Internationally, there is widespread inequality in accessibility of healthcare services between migrants and local residents, especially in the low- and middle-income countries [28], including in China. However, little is known about whether air pollution plays a role in the healthcare-seeking decisions of migrants despite extensive studies into the effects of sociodemographic factors (especially age and income), health insurance entitlements, and seriousness of symptom presentations [26, 29].

## Methods

This study adopted a cross-sectional design using data that are publicly available. No ethics approval was required.

### Data source

Health services data were extracted from the 2017 China Migrants Dynamic Survey (CMDS). The CMDS employed a stratified three-stage probability proportionate to size (PPS) sampling strategy to select study participants from the domestic migrant populations in all of the 31 provinces/regions in mainland China. Data were collected through household visits and face-to-face interviews over a one-month period in May 2017. The survey covered data relating to the socioeconomic characteristics (gender, age, education, distance of migration, sense of local belonging, weekly work hour, per capita annual household income) of study participants, their health status (illness/injury/unwell over the past two weeks, self-rated general health, hypertension, diabetes), availability of healthcare resources (social health insurance, distance to nearest health facility), and access to healthcare services (health education, medical services). A total of 169,993 individual responses were returned: only one respondent from each household was allowed. Details of the CMDS methods were published elsewhere (https://www.chinaldrk.org.cn).

The CMDS data were linked to the air pollution data published by the China National Environmental Monitoring Center (http://www.cnemc.cn/) according to the residential location of the study participants. The platform publishes real-time weather and air quality information, which include particulate matter (PM_2.5_ and PM_10_) and air quality index (AQI) at the municipality level in China. Multiple state controlling air sampling sites were set up in each city. Most of the monitoring sites were located in urban areas, with a small number in suburban and rural areas. Details of the environmental conditions around the sampling sites, monitoring methods and analyzers can be available in the previous literature [30]. The daily average was calculated based on the real-time hourly data captured, while the monthly average was calculated based on the daily average data for the purpose of this study.

The CMDS data were also linked to the atmospheric temperature data of the 310 municipalities in which the study participants resided. The atmospheric temperature data were extracted from the MERRA-2 database released by the National Aeronautics and Space Administration (NASA) in the US. We used the product-M2I6NPANA (doi: 10.5067/A7S6XP56VZWS), which records the temperature of different layers of air from the surface to 36,000 m high (a total of 42 layers) every six hours. Thermal inversion was defined as a positive temperature difference between the second layer (320 m) and the first layer (110 m) [31]. We calculated daily average of the thermal inversion strength over the four six-hour periods each day (from 6 pm of the previous day to 6 pm of the current day), and used the daily average to generate monthly average. Each municipality has multiple records of daily average strength of thermal inversion grouped by grids, with each grid covering a 50 × 60 km area. A mean daily value for each municipality was calculated by pooling the records of all relevant grids. A monthly average of the daily mean values was eventually generated for each municipality.

### Air quality data processing

We conducted further quality control on the published real-time hourly air quality data (hourly air quality index (AQI), PM_2.5_ and PM_10_ at the municipality level (*n* = 310) in April and May 2017). Referring to previous studies [32, 33], the original time series were transformed into z scores, and then the unrealistic points in the hourly time series were removed when concurrently satisfying three conditions: (1) an absolute z score larger than 4 ($\left|{Z}_{t}\right|>4$); (2) the increment of the z score between the current time and a previous time larger than 6 (${Z}_{t}-{Z}_{t-1}>6$); and (3) the ratio of the z score to its centred moving average of order 3 larger than 2 (${3Z}_{t}/({Z}_{t-1}+{Z}_{t}+{Z}_{t+1})>2$). The daily average of the above-mentioned indicators were then computed from the hourly data captured when more than 75% of the measurements (18 out of 24) in a day were valid data [33]. We calculated the monthly average of these indicators for each municipality using the daily average data in April and May 2017 for the purpose of this study.

#### Measurements

##### Dependent variable

The respondents who felt ill/injured/unwell (*n* = 10,051) over the past two weeks were included in data analyses. Visiting (Y = 0) or refraining from visiting (Y = 1) health facilities (hospitals, clinics, community health centers, and pharmacy retail outlets) [34] was treated as the dependent variable.

##### Independent variable

The association between air pollution and refraining from visiting health facilities was the major interest of this study. PM_2.5_ has been a preferred measurement of air quality in environmental health studies [35, 36]. In this study, we also used AQI and PM_10_ as an alternative indicator of air pollution.

Although the exact dates when the study participants intended to visit health facilities were not known, they fell within the period from 17 April to 31 May 2017. We used three time windows (April average, May average, and April-May average) to test the associations between the air pollution indicators and refraining from visiting health facilities.

We also categorized PM_2.5_ and AQI levels into air pollution grades, which align more closely with how people perceive [37, 38] and adjust their behaviors [39]. Specifically, the PM_2.5_ level was classified as normal (< 35 µg/m^3^) or abnormal (≥ 35 µg/m^3^) according to the air quality standards (GB3095-2012) in China. These standards have been widely accepted by the public [40]. AQI represents overall air quality considering all kinds of pollution, which was classified into five grades: good (0–50), moderate (51–100), unhealthy (101–150), very unhealthy (151–200), and hazardous (200+). The five categories were collapsed into three to correct the skewed distribution of data: good (0–50), moderate (51–100), unhealthy (> 100).

##### Instrumental variable

Thermal inversion intensity was adopted as an instrumental variable to overcome potential endogeneity. Thermal inversion is an unusual meteorological phenomenon where air temperature rises with height, which can hinder the diffusion of pollutants [41]. However, it does not directly affect human health and healthcare seeking behaviors, and has therefore become an instrumental variable in air pollution-related health studies [31, 42]. In this study, monthly average of thermal inversion intensity for each municipality in April, May, and April-May 2017 was calculated in line with the time window of air pollution indicators.

##### Control variable

Selection of the control variables followed the Anderson’s health service utilization model [43]. These included: (1) Predisposing factor - demographic characteristics (gender, age) and social status (education); (2) Enabling factor - distance of migration (inter-provincial, inter-municipal, inter-county), sense of local belonging, weekly work hours, exposure to health education, social health insurance, per capita annual household income (Yuan), and walking distance (minutes) to nearest healthcare facility; (3) Need factor - self-rated general health, hypertension and diabetes diagnosed by a medical doctor.

### Statistical analysis

The proportion of study participants who refrained from visiting health facilities when needed was calculated and compared between those with different characteristics using Chi-square tests.

Probit regression models were established to determine the effect of air pollution on refraining from visiting health facilities after adjustment for variations in the instrumental and control variables (see Supplementary Table for the coding scheme).

$$Y=a_0+a_1Air\;Pollution+a_j{Control}_j+\varepsilon$$

In the model, *Y* represents the probability of refraining from visiting health facilities. *Air Pollution* represents the air quality indicator of the municipality, while *Control*_*j*_ is a set of instrumental and control variables measured at the individual level (_*j*_). *ε* denotes the error term.

Since the intra-cluster (municipality) correlation coefficient (ICC) was low (0.076 < 0.1) indicating a lack of cluster effect [44], a single-level modeling approach was adopted despite the two levels of measurements. We established the probit regression models using April (Model One), May (Model Two), and April-May PM_2.5_ (Model Three), respectively, before introducing the instrumental variable (corresponding monthly thermal inversion intensity) into the models. We also tested the robustness of the findings by replacing PM_2.5_ with PM_10_ and AQI.

All statistical analyses were performed using Stata/MP 17.0. A two-side *p* value of lower than 0.05 was considered statistically significant.

## Results

### Characteristics of study participants

Slightly more than half (53.42%) of study participants were female. The vast majority were younger than 41 years (62.92%) and completed up to middle school education (42.13%). Inter-provincial (long distance) migration accounted for nearly half (46.84%) of the migrations. More than half (51.84%) of study participants worked more than 60 h a week. Over 90% enrolled in a basic medical insurance scheme and 83.52% could reach a health facility within 15 min, but 72.25% had a low sense of local belonging. Only 10.02% reported hypertension and/or diabetes, while 90.24% rated their general health as good (Table 1).

**Table 1:** Characteristics of study participants

Variables	Categories	Number	Proportion (%)
Sex	Male	4682	46.58
	Female	5369	53.42
Age (Year)	≤ 20	315	3.13
	21–40	6009	59.79
	41–60	3207	31.91
	> 60	520	5.17
Educational attainment	None	479	4.77
	Primary school	1845	18.36
	Middle school	4234	42.13
	High school or above	3493	34.75
Distance of migration	Inter-provincial	4708	46.84
	Inter-municipal	3291	32.74
	Inter-county	2052	20.42
Basic medical insurance	Uninsured	914	9.09
	Insured	9137	90.91
Per capita annual household income (Yuan)	≤ 10,000	1082	10.77
	10,001–20,000	3371	33.54
	20,001–30,000	2533	25.20
	30,001–40,000	1405	13.98
	> 40,000	1660	16.52
Weekly work hour	≤ 60 h	4841	48.16
	> 60 h	5210	51.84
Walking distance to nearest health facility (Minute)	< 15	8395	83.52
	≥ 15	1656	16.48
Sense of local belonging	High level	2789	27.75
	Low level	7262	72.25
Attending community health education over the past year	No	3414	33.97
	Yes	6637	66.03
Self-rated health	Poor	981	9.76
	Good	9070	90.24
Hypertension/Diabetes	No	9044	89.98
	Yes	1007	10.02

### Refraining from visiting health facilities

About 16% of study participants reported refraining from visiting health facilities when needed over the past two weeks. Those who were younger, obtained a higher level of education, migrated inter-provincially, had higher per capita annual household income, did not attend health education classes in the past year, had a low sense of local belonging, reported good self-related health, and did not have hypertension/diabetes were more likely to refrain from visiting health facilities when needed (Table 2).

**Table 2:** Incidence of refraining from visiting health facilities by study participants

Characteristics of study participants		Refraining from visiting health facilities			
		**Number**	**Incidence (%)**	**χ** ^**2**^	***P***
Sex	Male	781	16.68	1.269	0.260
	Female	851	15.85		
Age (Year)	≤ 20	66	20.95	17.165	0.001
	21–40	1025	17.06		
	41–60	473	14.75		
	> 60	68	13.08		
Educational attainment	None	75	15.66	21.679	< 0.001
	Primary school	253	13.71		
	Middle school	662	15.64		
	High school or above	642	18.38		
Distance of migration	Inter-provincial	810	17.20	6.761	0.034
	Inter-municipal	517	15.71		
	Inter-county	305	14.86		
Basic medical insurance	Uninsured	150	16.41	0.022	0.881
	Insured	1482	16.22		
Per capita annual household income (Yuan)	≤ 10,000	170	15.71	28.000	< 0.001
	10,001–20,000	470	13.94		
	20,001–30,000	423	16.70		
	30,001–40,000	246	17.51		
	> 40,000	323	19.46		
Weekly work hours	≤ 60 h	791	16.34	0.072	0.788
	> 60 h	841	16.14		
Walking distance to nearest health facility (Minute)	< 15	1361	16.21	0.024	0.878
	≥ 15	271	16.36		
Sense of local belonging	High level	517	18.54	15.013	< 0.001
	Low level	1115	15.35		
Attending community health education over the past year	No	643	18.83	25.638	< 0.001
	Yes	989	14.90		
Self-rated health	Poor	112	11.42	18.572	< 0.001
	Good	1520	16.76		
Hypertension/Diabetes	No	1516	16.76	18.315	< 0.001
	Yes	116	11.52		

### Association between air pollution and refraining from visiting health facilities

On average, the participants were exposed to 39.36 (SD = 11.76) µg/m^3^ PM_2.5_, 86.38 (SD = 34.03) µg/m^3^ PM_10_, and 70.84 (SD = 18.96) AQI in April-May 2017. Compared with the study participants who visited health facilities, those who refrained from visiting health facilities were exposed to higher levels of April-May PM_2.5_ (41.03 ± 11.05 vs. 39.03 ± 11.87, *p* < 0.001) and April-May PM_10_ (89.40 ± 32.07 vs. 85.80 ± 34.36, *p* < 0.001), and poorer April-May AQI (73.04 ± 17.96 vs. 70.41 ± 19.12, *p* < 0.001).

Air pollution was found to be a significant predictor of refraining from visiting health facilities in the probit regression models, with a consistent regression coefficient (β = 0.002, *p* < 0.001) for April-May (Model One), May (Model Two). An increase of one µg/m^3^ PM_2.5_ was associated with 0.2% points increase in the probability of refraining from visiting health facilities (Table 3).

Age, educational attainment, and distance of migration became insignificant predictors in some or all of the probit regression models; whereas, weekly working hours became a significant predictor after adjustment for variations in other control variables (Table 3).

**Table 3:** Probit regression models on refraining from visiting health facilities

Variables	β Coefficient (Standard Error)		
	Model One	Model Two	Model Three
PM_2.5_ (µg/m^3^) in April and May	0.002^c^ (0.000)		
PM_2.5_ (µg/m^3^) in April		0.002^c^ (0.000)	
PM_2.5_ (µg/m^3^) in May			0.001^c^ (0.000)
Sex: Male vs. Female	0.009 (0.007)	0.010 (0.007)	0.009 (0.007)
Age (Years): ≤20	1	1	1
21–40	-0.029 (0022)	-0.029 (0.022)	-0.028 (0.022)
41–60	-0.033 (0.023)	-0.033 (0.023)	-0.033 (0.023)
> 60	-0.041 (0.029)	-0.042 (0.029)	-0.040 (0.029)
Education: None	1	1	1
Primary school	-0.031 (0.020)	-0.034^a^ (0.020)	-0.028 (0.020)
Middle school	-0.024 (0.020)	-0.026 (0.020)	-0.021 (0.020)
High school or above	0.002 (0.021)	-0.004 (0.021)	0.002 (0.021)
Distance of migration: Inter-provincial	1	1	1
Inter-municipal	-0.009 (0.009)	-0.007 (0.009)	-0.010 (0.009)
Inter-county	-0.015 (0.010)	-0.011 (0.010)	-0.016 (0.010)
Basic medical insurance: Yes vs. No	0.004 (0.013)	0.005 (0.013)	0.004 (0.013)
Annual household income (Yuan): ≤10,000	1	1	1
10,001–20,000	-0.029^b^ (0.014)	-0.031^b^ (0.014)	-0.028^b^ (0.014)
20,001–30,000	-0.008 (0.015)	-0.011 (0.015)	-0.006 (0.014)
30,001–40,000	-0.009 (0.016)	-0.013 (0.016)	-0.006 (0.016)
> 40,000	0.002 (0.016)	-0.000 (0.016)	0.005 (0.016)
Weekly work hours	0.015^a^ (0.008)	0.014^a^ (0.008)	0.015^a^ (0.008)
Walking distance to nearest health facility (Minutes)	0.007 (0.010)	0.006 (0.010)	0.006 (0.010)
Sense of local belonging: Yes vs. No	-0.019^b^ (0.008)	-0.019^b^ (0.008)	-0.019^b^ (0.008)
Attending community health education over the past year: Yes vs. No	-0.037^c^ (0.008)	-0.037^c^ (0.008)	-0.038^c^ (0.008)
Self-rated health: Good vs. Poor	0.040^c^ (0.015)	0.041^c^ (0.015)	0.040^c^ (0.015)
Hypertension/Diabetes: Yes vs. No	-0.040^c^ (0.014)	-0.040^c^ (0.014)	-0.040^c^ (0.014)

^a,^^b,^^c^denote statistically significant difference at the 10%, 5% and 1% significance level

The introduction of the instrumental variable (monthly average of thermal inversion intensity) to the probit regression models did not change the direction and degree of association between April-May PM_2.5_ and refraining from visiting health facilities (β = 0.018, *p* < 0.001), despite its significant association with April-May PM_2.5_ (*p* < 0.001). Similarly, the effects of other significant predictors remained largely unchanged (Table 4).

**Table 4:** Ivprobit models: an estimate of air pollution on refraining from visiting health facilities

Predictors	Effect of instrument variable on PM_2.5_ (April-May)	Effect of PM_2.5_ (April-May) on refraining from visiting health facilities
PM_2.5_		0.018^c^ (0.002)
Thermal inversion (instrument variable)	8.298^c^ (0.133)	
Sex: Male vs. Female	-0.101 (0.202)	0.046 (0.031)
Age (Years): ≤20	1	1
21–40	-0.681 (0.576)	-0.129 (0.083)
41–60	-0.730 (0.604)	-0.147^a^ (0.088)
> 60	-1.175 (0.758)	-0.187 (0.115)
Education: None	1	1
Primary school	0.011 (0.517)	-0.144^a^ (0.081)
Middle school	0.467 (0.510)	-0.123 (0.079)
High school or above	1.391^c^ (0.532)	-0.046 (0.083)
Distance of migration: Inter-provincial	1	1
Inter-municipal	0.650^c^ (0.230)	-0.045 (0.035)
Inter-county	1.332^c^ (0.273)	-0.085^b^ (0.043)
Basic medical insurance: Yes vs. No	2.533^c^ (0.347)	-0.007 (0.054)
Annual household income (Yuan): ≤10,000	1	1
10,001–20,000	-1.130^c^ (0.353)	-0.123^b^ (0.055)
20,001–30,000	-1.476^c^ (0.376)	-0.040 (0.058)
30,001–40,000	-1.069^b^ (0.424)	-0.049 (0.065)
> 40,000	-0.381 (0.420)	-0.005 (0.064)
Weekly work hours	-0.597^c^ (0.209)	0.066^b^ (0.032)
Walking distance to nearest health facility (Minutes)	-0.316 (0.267)	0.039 (0.041)
Sense of local belonging: Yes vs. No	-0.643^c^ (0.229)	-0.082^b^ (0.035)
Attending community health education over the past year: Yes vs. No	0.347 (0.213)	-0.142^c^ (0.032)
Self-rated health: Good vs. Poor	0.559 (0.376)	0.171^c^ (0.062)
Hypertension/Diabetes: Yes vs. No	-0.403 (0.366)	-0.169^c^ (0.060)
Constant	31.150^c^ (0.905)	-1.447^c^ (0.161)
First-stage F statistic	200.09	
Wald test for endogeneity		26.68^c^

^a,^^b,^^c^denote statistically significant difference at the 10%, 5% and 1% significance level

### Robustness test

To test the robustness of the findings, we replaced April-May PM_2.5_ (continuous measurement) with April-May PM_10_ (continuous measurement, Model Four), the categorical indicator of April-May PM_2.5_ (Model Five), or the categorical indicator of April-May AQI (Model Six). The results remained robust (Table 5).

**Table 5:** Effects of different air pollution indicators on refraining from visiting health facilities

Variables	Probit Model Four	Probit Model Five	Probit Model Six
PM_10_	0.0005^c^ (0.000)		
PM_2.5_: ≤35		1	
PM_2.5_: >35		0.049^c^ (0.008)	
AQI: 0–50			1
AQI: 51–100			0.049^c^ (0.010)
AQI: >100			0.047^c^ (0.016)
Sex: Male vs. Female	0.008 (0.007)	0.009 (0.007)	0.009 (0.007)
Age (Years): ≤20	1	1	1
21–40	-0.030 (0.022)	-0.027 (0.022)	-0.024 (0.022)
41–60	-0.034 (0.023)	-0.032 (0.023)	-0.028 (0.023)
> 60	-0.042 (0.029)	-0.040 (0.029)	-0.036 (0.028)
Education: None	1	1	1
Primary school	-0.029 (0.020)	-0.030 (0.020)	-0.030 (0.020)
Middle school	-0.021 (0.020)	-0.023 (0.020)	-0.020 (0.020)
High school or above	0.002 (0.021)	0.000 (0.021)	0.005 (0.021)
Distance of migration: Inter-provincial	1	1	1
Inter-municipal	-0.011 (0.009)	-0.007 (0.009)	-0.008 (0.009)
Inter-county	-0.014 (0.010)	-0.011 (0.010)	-0.013 (0.010)
Basic medical insurance: Yes vs. No	0.005 (0.013)	0.006 (0.013)	0.005 (0.013)
Annual household income (Yuan): ≤10,000	1	1	1
10,001–20,000	-0.027^b^ (0.013)	-0.031^b^ (0.014)	-0.029^b^ (0.014)
20,001–30,000	-0.005 (0.014)	-0.011 (0.015)	-0.008 (0.015)
30,001–40,000	0.008 (0.016)	-0.012 (0.016)	-0.007 (0.016)
> 40,000	0.008 (0.016)	-0.001 (0.016)	0.005 (0.016)
Weekly work hours	0.013 (0.008)	0.016^b^ (0.008)	0.013^a^ (0.008)
Walking distance to nearest health facility (Minutes)	0.005 (0.010)	0.008 (0.010)	0.006 (0.010)
Sense of local belonging: Yes vs. No	-0.021^b^ (0.008)	-0.020^b^ (0.008)	-0.019^b^ (0.008)
Attending community health education over the past year: Yes vs. No	-0.038^c^ (0.008)	-0.037^c^ (0.008)	-0.036^c^ (0.008)
Self-rated health: Good vs. Poor	0.041^c^ (0.015)	0.040^c^ (0.015)	0.040^c^ (0.015)
Hypertension/Diabetes: Yes vs. No	-0.040^c^ (0.014)	-0.040^c^ (0.014)	-0.040^c^ (0.014)

^a,^^b,^^c^denote statistically significant difference at the 10%, 5% and 1% significance level

## Discussion

Although local healthcare institutions can provide better care for the common and frequently-occurring health problems of migrants [45], we found that more than 16% of the migrant respondents refrained from visiting health facilities when needed over a two-week period. This level of refraining appears to be lower than that (63%) revealed in a survey of domestic migrants in Guangdong province of China in 2012 [46]. It is also lower than what was found in the general population of Sweden (24%) [47] and the female undocumented immigrants in the Netherlands (56%) [48]. It is important to note that many previous studies considered purchase of medicines from community pharmacy retail outlets as part of self-medication, resulting in a much higher percentage of migrants refraining from seeking healthcare [49]. Self-medication is the top strategy for managing illness conditions in migrant workers in some countries [50]. In our study, however, pharmacies were considered as one of the health facilities, because the study participants needed to go outside of their houses (being exposed to polluted air) to purchase medicines from pharmacies. Self-medication was reported by almost one quarter (24.32%) of the participants in our study.

Our study offers some new insight into the adverse health effects of air pollution. We found that exposure to ambient air pollutants (PM_2.5_, AQI, and PM_10_) is a significant predictor of refraining from visiting health facilities. Other factors that are associated with refraining from visiting healthcare include long-distance (inter-provincial) migration, overwork, good self-rated health, low sense of local belonging, a lack of health education, and absence of hypertension/diabetes.

### Negative effect of air pollution on seeking healthcare

We found that air pollution is associated with refraining from visiting health facilities in the domestic migrants in China. A large number of previous studies have revealed negative consequences of air pollution on human health [51], including in the patients with asthma, chronic obstructive pulmonary disease, and cardiovascular diseases [17]. Epidemiological and controlled exposure studies show that anyone can suffer from respiratory symptoms and lung function impairment resulting from exposure to air pollution [52]. People are becoming increasingly concerned about the impacts of air pollution on their health and daily lives [53]. Air pollution reduces people’s willingness and frequency of travelling [54]. With easy access to air quality information through the modern information and communication technologies, individuals can easily adjust their health and healthcare-seeking behaviors in line with the level of perceived health risks of air pollution [55]. A high level of awareness of air pollution and its harm is associated with consumer actions relating to preventive and curative health measures [21, 56]. These may lead to avoidance of or shortened exposure to external environments [57]. Empirical evidence shows that the public tend to over-estimate the intensity and hazard of air pollution [58], causing excessive anxiety and reduced well-being [40]. The widespread concern about air pollution can also trigger social media sensation [59], further exacerbating people’s responses. Those who feel threatened by air pollution may refrain from visiting health facilities when needed.

### Overwork and refraining from visiting health facilities

People usually migrate to other places in seeking better job opportunities. However, the jobs taken by migrants are often characterized by long working hours, inflexible work schedules, and a lack of job security, which can hinder their access to healthcare services [60]. Our study provides further evidence to support the association between overwork and refraining from visiting health facilities in the migrant populations. Migrants tend to take heavy workloads, squeezing out of free time for leisure and social activities. In China, there have been increasing concerns about the suboptimal working conditions of domestic migrants, as well as the lower level of social welfare and healthcare entitlements of domestic migrants relative to long-term local residents [61, 62].

### Low social integration and refraining from visiting health facilities

Our study shows that a low sense of local belonging and long-distance (inter-provincial) migration are predictors of refraining from visiting health facilities. Migrants, in particular those who have a temporary or short-term living arrangement, face great challenges in adapting to the new environments and obtaining local social support [63, 64]. As a result, the continuity of healthcare services needed by migrants can be compromised. Disruption of the existing social networks often makes the migrants feel strange to the health care system of the migrant destinations [65], jeopardizing their ability to navigate through the often complex health system [62]. Adding to the complexity are language barriers and socioeconomic discriminations [66–68].

### Health education and refraining from visiting health facilities

Health education is key to increasing health literacy, which is usually linked to high use of preventive care [69]. Our study found that the domestic migrants who received community health education are less likely to refrain from visiting health facilities. This result is consistent with the findings of other studies. Inadequate health literacy is associated with low levels of awareness of healthcare needs and the availability of preventive interventions [70]. A cross-sectional study of migrant workers in China found that health education is associated with high self-consciousness of seeking healthcare [4].

### Self-related health, chronic conditions, and refraining from visiting health facilities

Felt health needs play an important role in healthcare seeking decisions in the migrants, according to the findings of our study and some others. We found that those who perceived poor health and suffered from chronic conditions are less likely to refrain from visiting health facilities than others. Shi and colleagues argued that the relatively young and healthy migrants have high capability of ignoring and overcoming health problems [71]. By contrast, the migrants with moderate and severe symptoms have a more urgent feeling of needs to seek medical care [72].

## Strengths and limitations

This study makes a significant contribution to the literature regarding the health hazards associated with air pollution. The sample size is large. We used three air pollution indicators to determine the association between air pollution and refraining from visiting health facilities when needed. An instrumental variable (strength of thermal inversion) was added to the probit regression modelling to address potential endogeneity.

There are several limitations of this study. First, the dataset used in the study did not tell what illness or how serious the illness conditions were when the study participants refrained from visiting health facilities, although self-rated general health was measured. Second, this study adopted a cross-sectional design, which prevents us from drawing causal conclusions. However, it is unlikely that healthcare seeking behaviors would have a significant impact on air pollution. In China, the vast majority of populations can find a health facility within a 15-minute walking distance. Although we used an instrumental variable to address the endogeneity issue, further studies are recommended to explore the underlying mechanisms of the association between air pollution and refraining from visiting health facilities.

## Conclusions and policy implications

This study aimed to determine the association between air pollution and refraining from visiting health facilities in the domestic migrant populations in China. We found that air pollution is a significant predictor of refraining from visiting health facilities. This finding was validated using multiple air pollution indicators, multivariate regression modelling, and inclusion of an instrumental variable.

In addition to air pollution, domestic migrants in China can also be deterred from seeking healthcare due to inequalities in socioeconomic arrangements. Significant regional disparities exist in China, despite the unprecedented economic development over the past few decades [73]. Regional economic disparities are the key driver of population migration, which is often accompanied by social discrimination against migrants, inequality in welfare entitlements and a low sense of local belonging of the migrants [63]. Domestic migrants in China have made a great contribution to China’s economic miracle. Governmental policies should pay increasing attention to the wellbeing of the migrant populations, fostering better social integration.

## Supplementary Information

**Additional file 1:** **Supplementary Table.**Definition and coding of variables.

## Data Availability

The datasets used during the current study are publicly available. Health services data was available from China Migrant Population Service Center [https://www.chinaldrk.org.cn]. Air quality data was available from the China National Environmental Monitoring Center [http://www.cnemc.cn/]. In addition, the atmospheric temperature data was available in https://gmao.gsfc.nasa.gov/.

## Abbreviations

PM_2.5_: Particulate matter with aerodynamic diameter ≤ 2.5 μm
PM_10_: Particulate matter with aerodynamic diameter ≤ 10 μm
AQI: Air Quality Index
CMDS: China Migrants Dynamic Survey
