# Supplementary material for: Air pollution and refraining from visiting health facilities: a cross-sectional study of domestic migrants in China
Source: BMC Public Health. 2022 Nov 2;22:2007. doi: 10.1186/s12889-022-14401-4 (PMC9628112; doi:10.1186/s12889-022-14401-4)
Supplement: Supplementary file 1 — Additional file 1: Supplementary Table.Definition and coding of variables. [file 12889_2022_14401_MOESM1_ESM.docx]

| **Supplementary Table. Definition and coding of variables** | | | |  |
| --- | --- | --- | --- | --- |
| **Category** | **Variable** | **Definition** | **Coding assignment** | |
| Dependent variable | Refraining from visiting health facilities | Where did you first go for medical attention when you were ill/injured or felt unwell? | 1=Refraining from visiting health facilities  0=Visiting health facilities | |
| Independent variable | PM_2.5_ | PM_2.5_ concentration (μg/m^3^) in April and May 2017 | | |
|  | PM_10_ | PM_10_ concentration (μg/m^3^) in April and May 2017 | | |
|  | AQI | Air quality index (0-200) in April and May 2017 | | |
| Predisposing factor | Sex | Biological sex | 1=Male  0=Female | |
|  | Age | Age groups (years) | 1= ≤20  2=21-40  3=41-60  4=>60 | |
|  | Educational attainment | Highest level of formal education | 1=None  2=Primary school  3=Middle school  4=High school or above | |
| Enabling factor | Distance of migration | Distance from original residence to migration destination | 1=Inter-provincial  2=Inter-municipal  3=Inter-county | |
|  | Sense of local belonging | A feeling as a local person | 0=Low level  1=High level | |
|  | Weekly working hours | How many hours did you work this week? | 0=≤60  1=>60 | |
|  | Health education | Have you received any health education in your local community last year? | 0=No  1=Yes | |
|  | Basic medical insurance | Have you enrolled in a basic medical insurance program? | 0=No  1=Yes | |
|  | Household income | Per capita annual household income (Yuan) | 1=≤10000  2=10001-20000  3=20001-30000  4=30001-40000  5=>40000 | |
|  | Distance to nearest health facility | Walking distance (minutes) to the nearest health facility | 0=Within 15 minutes  1=15 minutes or more | |
|  | Self-rated general health | How is your health in general? | 0=Poor  1=Good | |
|  | Hypertension/Diabetes | Have you been diagnosed with hypertension or diabetes? | No = 0; Yes = 1 | |
| Instrumental variable | Thermal inversion intensity | Atmospheric temperature in the second layer (320 meters) minus that in the first layer (110 meters) | | |
